# CD3^+^CD8^+^CD28^−^ T Lymphocytes in Patients with Lupus Nephritis

**DOI:** 10.1155/2016/1058165

**Published:** 2016-06-30

**Authors:** Marcelina Żabińska, Magdalena Krajewska, Katarzyna Kościelska-Kasprzak, Marian Klinger

**Affiliations:** Department and Clinic of Nephrology and Transplantation Medicine, Faculty of Postgraduate Medical Training, Wroclaw Medical University, Borowska 213, 50-556 Wroclaw, Poland

## Abstract

The results of studies on the CD3^+^CD8^+^CD28^−^ cells in SLE are inconsistent since several analyses describe CD3^+^CD8^+^CD28^−^ as either immunosuppressive or cytotoxic. The aim of this study is to inquire whether the quantitative changes of CD3^+^CD8^+^CD28^−^ T lymphocytes subpopulation are related to the clinical status of patients with lupus nephritis. Evaluation of Foxp3 expression on CD3^+^CD8^+^CD28^−^ cells may shed some light on functional properties of these cells. 54 adult SLE patients and 19 sex and age matched healthy volunteers were enrolled in the study. There were 15 patients in inactive (SLEDAI ≤ 5) and 39 in active (SLEDAI > 5) phase of disease. We determined absolute count of CD3^+^CD8^+^CD28^−^ and CD3^+^CD8^+^CD28^−^Foxp3^+^ subpopulations by flow cytometry. We observed a statistically significant increase in absolute count and percentage of CD3^+^CD8^+^CD28^−^ in SLE patients compared to HC (*p* < 0.001). Moreover there was significant positive correlation between increasing absolute count of CD3^+^CD8^+^CD28^−^ cells and disease activity measured by SLEDAI (*r*s = 0.281, *p* = 0.038). Active LN patients had increased absolute count of CD3^+^CD8^+^CD28^−^ cells compared to HC. Positive correlation of CD3^+^CD8^+^CD28^−^ number with disease activity, and lack of Foxp3 expression on these cells, suggests that CD3^+^CD8^+^CD28^−^ lymphocytes might be responsible for an increased proinflammatory response in the exacerbation of SLE.

## 1. Introduction

T  CD8^+^ cells play a key role in the recognition and removal of cells infected by intracellular pathogens [1] and also in antitumor response [2]. Binding of T  CD8^+^ surface receptor TCR and MHC-I-bound antigen, found on the professional antigen presenting cell's (pAPC) surface, leads to T  CD8^+^ activation [3]. Since stimulation only through the TCR receptor is unable to maintain optimum T  CD8^+^ activation, the second costimulatory signal is essential for full activation and survival of these cells [4]. The best known costimulatory signal is provided by the interaction of CD28 molecules presented on the T lymphocyte as well as the CD86 and CD80 molecules expressed on the pAPC's surface [3]. Adequate signal power delivered to naive T  CD8^+^ results in the proliferation and differentiation of two cell types. One of these is cytotoxic T lymphocytes (CTL), which after reaching maturity and fulfilling their effector function undergo apoptosis. The second type is T  CD8^+^ memory cells, both central and effector. Their continuous presence in the circulation is essential to control another potential exposure to the same antigen in a faster and more effective way [5].

Under chronic antigen stimulation, repeated cycles of activation occur and lead to progressive and irreversible reduction in CD28 molecule expression on the lymphocyte surface. This results in accumulation of “highly antigen-experienced” T-cell with CD8^+^CD28^−^ phenotype characterized by extremely shortened telomeres [6].

There is a close relationship between CD28 molecules presence and degeneration of the telomeres/telomerase. Telomerase activity is necessary for cell proliferation, cytokines and chemokines production, and antiviral activity. However, lack of CD28 molecules leads to loss of ability to increase telomerase activity in activated cells. Maintaining the presence of CD28 molecule through gene transduction* in vitro* slows down the “immune aging” and improves the efficiency of the immune system [7]. Telomeres are set up on chromosomes ends and ensure their stability. Unprotected chromosomes ends are exposed to a high risk of degradation. Such degradation processes lead to the genetic information loss and cell death [8]. This process functions as a mitotic clock, while telomere length represents the number of cell divisions [9].

Relationship has been demonstrated between the average telomere length in peripheral blood cells and autoimmune diseases, such as SLE [10, 11], rheumatoid arthritis [12], systemic sclerosis (SSc) [13], ANCA-associated vasculitis (AAV) [14], psoriasis, and atopic dermatitis [15].

It is now believed that one of the major causes of abnormal immune response is the telomere properties dysfunction that leads to autoimmunity [16]. Nonetheless some authors did not confirm the relationship between telomere dysfunction and the development of SLE [17]. It has been also observed that the loss of CD28 is associated with increased surface expression of the CD57 molecule. T CD8^+^CD28^−^ cells (CD8^+^CD57^+^) are referred to as antigen-specific, terminally differentiated, but also as functionally competent memory or effector T-cells which have gone through many cycles of cell division. Decrease or even loss of telomerase 5 activity and a low level of expression of genes involved in cell cycle regulation are characteristics for these cells. T  CD8^+^CD28^−^ (CD8^+^CD57^+^) cells have often limited ability to proliferate upon stimulation and it is believed that they have reached a state of “replicative old age” or “clonal exhaustion” [3, 18].

Data on the sensitivity to apoptosis of CD8^+^CD28^−^ (CD8^+^CD57^+^) lymphocytes are contradictory. Some of researchers [19, 20] argue that these cells are highly susceptible to activation induced apoptosis. This hypothesis is evidenced by increased expression of Fas and caspase-3 and decreased expression of antiapoptotic molecules such as survivin or heat shock protein 27 (HSP 27). Others maintain that T CD8^+^CD28^−^ (CD8^+^CD57^+^) lymphocytes show a high resistance to apoptosis and thus gradually accumulate throughout life [21, 22].

Most of autoimmune diseases are associated with an increase in T CD8^+^CD28^−^ (CD8^+^CD57^+^) cells, which exhibit the highly cytotoxic activity and can be related to more severe manifestations of the disease. Quantitative changes in CD8^+^CD57^+^ population were observed, among others, in multiple sclerosis [23], type 1 diabetes [23], Graves' disease [24], and rheumatoid arthritis [25]. The decreased number of CD8^+^CD28^−^ T-cells correlates with clinical response to abatacept in patients with rheumatoid arthritis [26]. Some researchers have reported that lymphocytes with CD8^+^CD28^−^ phenotype show regulatory properties. There are analyses which confirmed the presence of Foxp3 molecule in these cells [27, 28]; however there are analyses which showed no expression of this factor [29, 30]. Moreover, aside from lack of Foxp3 expression, markers characteristic for cytotoxic cells, such as granzyme A or perforin, were detected on CD8^+^CD28^−^ surface [31].

So far, few studies addressing the size of the CD8^+^CD28^−^ subpopulation in patients with SLE have been conducted. It has been shown that the number of CD8^+^CD28^−^ cells might be reduced or unchanged as compared to the control group [32, 33].

The main goal of the study was to investigate whether the quantitative changes of CD8^+^CD28^−^ T lymphocytes subpopulation are related to clinical status of patients with LN. Detection of Foxp3 molecule expression in CD8^+^CD28^−^ cells may shed some light on functional properties of these cells.

## 2. Material

54 adult SLE patients (96.3% female, mean age 36.5 ± 13.7) in the various phases of disease activity were enrolled into the study. Disease activity at the time of evaluation was scored according to the Systemic Lupus Erythematosus Disease Activity Index (SLEDAI) [34]. Patients were divided into two groups according to their SLEDAI score and there were 15 patients in inactive (SLEDAI ≤ 5) and 39 in active (SLEDAI > 5) phase of disease [35]. Additionally patients were split into two groups according to their renal SLEDAI (refers to the total of all renal components used to calculate the SLEDAI) and there were 14 (rSLEDAI < 4) and 40 (rSLEDAI 4–16) patients, respectively. Demographic characteristics and clinical data of the study group have been presented in Table 1. Additionally, 19 sex and age matched healthy volunteers (89.5% female, mean age 38.3 ± 14.1) served as the control group.

Patients were treated at the Department of Nephrology and Transplantation Medicine, Wroclaw Medical University, in accordance with the current guidelines for lupus nephropathy.

Patients with newly diagnosed renal disease in course of SLE used steroids infusion, with the conversion to the oral steroids at tapering doses, or were given only oral steroids. Furthermore, the immunosuppressive therapy included cyclophosphamide, azathioprine, cyclosporin A, mycophenolate mofetil, and chloroquine. In order to maintain remission, patients used oral steroids or oral steroids combined with mycophenolate mofetil or azathioprine.

Exclusion criteria of the study were presence of an active malignancy and inflammatory processes.

The study was approved by the Wroclaw Medical University Bioethics Committee.

## 3. Methods

### 3.1. Determination of CD3^+^CD8^+^CD28^−^ Subpopulation

**Figure 1:**
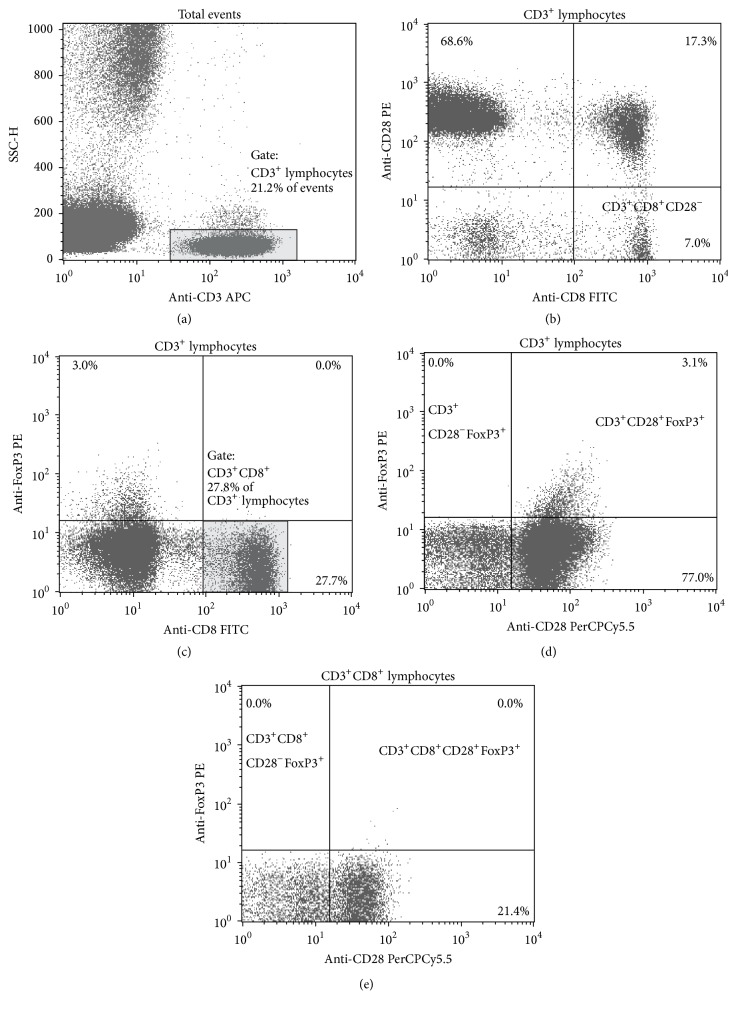
Flow cytometry analysis of CD3^+^CD8^+^CD28^−^ and CD3^+^CD8^+^CD28^−^Foxp3^+^ subpopulations. Gating strategy: (a) SSC versus anti-CD3 APC fluorescence plot. CD3^+^ lymphocytes are shown in the gate. (b) Anti-CD28 PE versus anti-CD8 FITC fluorescence plot. CD3^+^CD8^+^CD28^−^ cells are shown in lower right part of the chart. (c) Anti-FoxP3 PE versus anti-CD8 FITC fluorescence plot. CD3^+^CD8^+^ cells are shown in the gate. (d) Anti-FoxP3 PE versus anti-CD28 PerCPCy5.5 fluorescence plot. Foxp3 expression is present on non-CD8^+^ cells in upper right part of the chart. (e) Anti-FoxP3 PE versus anti-CD28 PerCPCy5.5 fluorescence plot. Lack of Foxp3 expression is shown on CD3^+^CD8^+^CD28^−^ cells in upper left part of the chart.

300 *μ*L of heparinized blood was stained with 20 *μ*L of the following antibodies: anti-CD3APC, anti-CD8FITC, and anti-CD28PE. All were purchased from Becton Dickinson (BD, San Jose, California, USA). After 30 minutes of incubation at 4°C in the dark, the red blood cells were lysed with BD FACS Lysing Solution (Becton Dickinson). The cells were washed twice with PBS 2% FBS and flow cytometry-analyzed (Figures 1(a) and 1(b)).

The measurement was accompanied with BD multitest TBNK (Becton Dickinson) for absolute cell count determination. For each sample, the absolute cell count of the population of CD3^+^CD8^+^CD28^−^ and their percentage in the population of T-cells were determined in relation to the number of CD3^+^ lymphocytes.

### 3.2. Determination of CD3^+^CD8^+^CD28^−^Foxp3^+^ Subpopulation

300 *μ*L of heparinized blood was stained with 20 *μ*L of the following antibodies: anti-CD3APC, anti-CD8FITC, and anti-CD28PerCPCy5.5. All were purchased from Becton Dickinson (BD, San Jose, California, USA). After 30 minutes of incubation at 4°C in the dark, the red blood cells were lysed with BD FACS Lysing Solution (Becton Dickinson). The cells were washed with PBS 2% FBS and permeabilized with the Fixation/Permeabilization Concentrate (eBioscience) in Fixation/Permeabilization Diluent (eBioscience) for 30 minutes at 4°C in the dark. After two washing steps in Permeabilization Buffer (eBioscience) the cell pellet was stained with 5 *μ*L of Anti-Human Foxp3 PE clone 236A/E7 (eBioscience, San Diego, CA, USA) for 30 minutes at 4°C in the dark. The samples were then washed twice in Permeabilization Buffer (eBioscience) and flow cytometry-analyzed (Figures 1(a)−1(e)). For each sample, the absolute cell number of CD3^+^CD8^+^CD28^−^Foxp3^+^ and their percentage in the population of T-cells were determined in relation to the number of CD3^+^ lymphocytes.

### 3.3. Statistical Analysis

The experimental and clinical data were combined and statistically analyzed using STATISTICA 10 software. The results of statistical analysis are presented with interquartile range. Correlation analysis was performed using the Spearman procedure. The Mann-Whitney *U* test (for independent samples) was applied, and differences with *p* less than 0.05 were considered statistically significant.

## 4. Results

The parameters measured regarding CD3^+^CD8^+^CD28^−^ cells were the percentage of the total T  CD3^+^ lymphocytes population and the absolute number of CD3^+^CD8^+^CD28^−^ cells in whole blood (values given per microliter). All blood samples revealed the presence of CD3^+^CD8^+^CD28^−^ cells. Statistically significant differences in both the percentage of CD3^+^CD8^+^CD28^−^ cells and their absolute numbers between the study group and the control group have been demonstrated. Patients with SLE presented significantly higher absolute count and percentage of CD3^+^CD8^+^CD28^−^ cells compared to HC (*p* < 0.001). Study group had more than three times higher absolute number (*p* < 0.001) and more than two times higher percentage of CD3^+^CD8^+^CD28^−^ cells (*p* < 0.001) compared to the control group.

**Figure 2:**
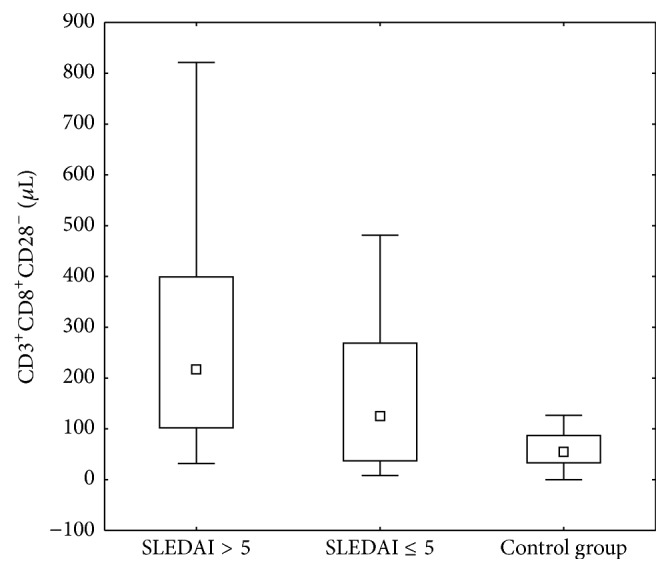
Comparison of the absolute number of CD3^+^CD8^+^CD28^−^ cells in the patients divided into two groups according to disease activity measured by SLEDAI scale and the control group.

**Figure 3:**
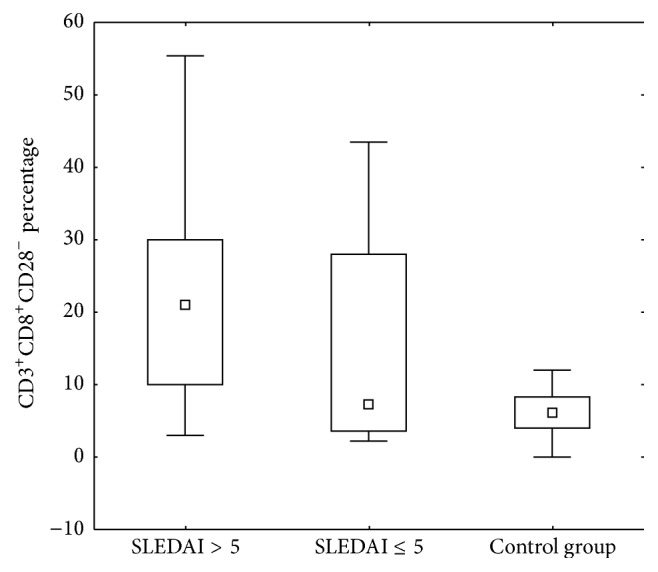
Comparison of the percentage of CD3^+^CD8^+^CD28^−^ cells in the patients divided into two groups according to disease activity measured by SLEDAI scale and the control group.

Additionally, variability in the number of CD3^+^CD8^+^CD28^−^ lymphocytes depending on the activity of the disease measured by SLEDAI scale has been observed (Figures 2 and 3).

The lower percentage of these cells was seen in the group with inactive disease compared to active LN patients (*p* = 0.022). There was also a significant difference in the absolute number of CD3^+^CD8^+^CD28^−^ cells, which was characterized by a lower frequency in patients with low disease activity compared to the group with high activity (*p* = 0.039).

Statistical analysis showed a significantly higher percentage and absolute values of CD3^+^CD8^+^CD28^−^ in patients with high disease activity compared with the control group (*p* < 0.001). Interestingly, the percentages and absolute count of CD3^+^CD8^+^CD28^−^ cells did not differ significantly between patients with inactive disease and the control group (Figure 3).

There were no significant differences in the percentage and absolute count of CD3^+^CD8^+^CD28^−^ between the groups according to rSLEDAI, but in the group with active nephritis (rSLEDAI 4–16) higher values of CD3^+^CD8^+^CD28^−^ have been observed compared to the group with inactive LN.

**Figure 4:**
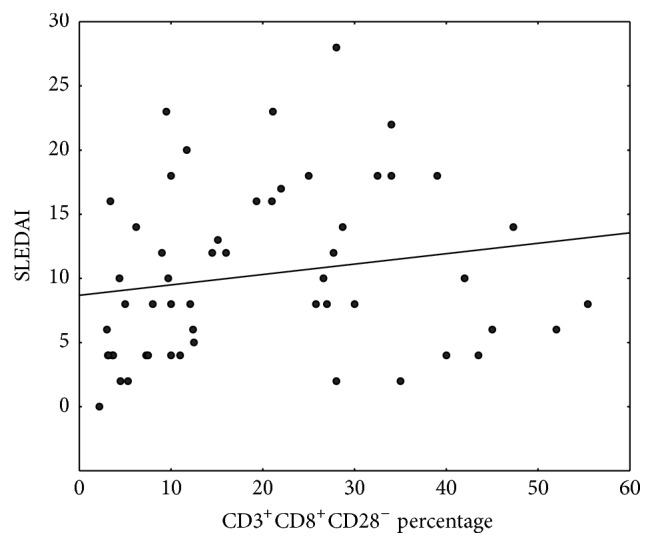
Correlation between the SLEDAI and CD3^+^CD8^+^CD28^−^ cells percentage.

Significant (*p* = 0.038) positive correlation between increasing percentage of CD3^+^CD8^+^CD28^−^ and disease activity measured by SLEDAI (correlation coefficient 0.281) was also demonstrated (Figure 4).

Lack of Foxp3 expression on CD3^+^CD8^+^CD28^−^ cells in any of the tested blood samples was observed (Figure 1(e)).

## 5. Discussion

There are conflicting reports concerning Foxp3 expression on CD3^+^CD8^+^CD28^−^ lymphocytes. Some of researchers indicate lack of this factor [29, 30] or, on the contrary, others have reported presence of this molecule [27, 28] in T CD8^+^CD28^−^ cells. In our study, there was no expression of Foxp3 in CD3^+^CD8^+^CD28^−^ cells in any of the tested blood samples, both in the control and in the study group. The potential methodological error regarding Foxp3 detection was eliminated as presence of Foxp3 molecule was demonstrated on non-CD8^+^ cells (Figure 1(d)). The results of our work suggest nonsuppressive and nonregulative properties of the CD3^+^CD8^+^CD28^−^ subpopulation [36].

In the present study almost three times higher number of CD3^+^CD8^+^CD28^−^ lymphocytes in the study group compared with the control group was demonstrated. Most of autoimmune diseases are associated with an increase in T CD8^+^CD28^−^ (CD8^+^CD57^+^) cells which exhibit cytotoxic properties and can play an active role in the autoimmune response [36]. The majority of literature reports indicate an increased number of these cells in autoimmune diseases and define them as a cytotoxic subpopulation, having a negative impact on the development of the immune response. Quantitative changes of a CD8^+^CD28^−^ (CD8^+^CD57^+^) lymphocytes population have been observed in autoimmune diseases such as multiple sclerosis [23], type 1 diabetes [23], Graves' disease [24], and rheumatoid arthritis [25]. Only a few literature reports regarding assessment of CD3^+^CD8^+^CD28^−^ lymphocyte in SLE presented different results. In one publication, authors showed no significant differences in the percentage of these cells in the PBMC from patients with SLE compared to healthy controls. However, their data showed that three patients with SLE had high levels of CD3^+^CD8^+^CD28^−^ lymphocytes, which is in line with our findings. Additional analysis demonstrated that two of these patients had active disease and that another one was inactive, but analysis of these data revealed that there was no significant association between the levels of CD3^+^CD8^+^CD28^−^ cells and disease activity [32]. The second publication demonstrated lower absolute number of CD3^+^CD8^+^CD28^−^ cells in patients with SLE than in healthy controls although no significant difference was found. However, when authors evaluated the distribution of CD28 molecule within the CD8 T-cell population, the CD3^+^CD8^+^CD28^−^ T-cell population was significantly lower in patients with SLE compared to healthy individuals [33]. Moreover, authors found no association between the absolute numbers of CD3^+^CD8^+^CD28^−^ T-cell population and SLEDAI [33].

Under the influence of chronic antigen stimulation in SLE repeating cycles of activation, stimulation and proliferation lead to progressive and irreversible reduction in expression of CD28 molecules on the surface of cells [6]. The result is the accumulation of “antigen-experienced” T-cell phenotype CD8^+^CD28^−^. This observation was confirmed in our study. It is also suggested that persistent antigenic stimulation is accompanied by abnormal apoptosis of CD3^+^CD8^+^CD28^−^ [22, 37] which may be confirmed by increased number of these cells in our analysis. Similar results were observed in patients with HIV infection who also have chronic activation of T lymphocytes, particularly in the late stages of infection [37]. In the present study a significant correlation between the number of CD3^+^CD8^+^CD28^−^ cells and disease activity measured by SLEDAI scale was demonstrated. In patients with active disease we observed almost twice the number of these cells compared to patients with inactive SLE. Furthermore, the number of CD3^+^CD8^+^CD28^−^ cells in patients with inactive disease did not differ from the control group; consequently it was increased only in patients with active disease. This observation indicates that the accumulation of cells with a phenotype of CD3^+^CD8^+^CD28^−^ is linked to the exacerbation of disease activity. Our analysis is the first study which has proved such correlation. The relationship between the number of CD3^+^CD8^+^CD28^−^ cells and SLEDAI makes it an attractive target for research in SLE. We believe that further tests in larger groups of patients are required to fully elucidate the mechanisms involved in pathogenesis of the disease, as well as nature of CD3^+^CD8^+^CD28^−^ lymphocytes.

## 6. Limitations of the Study

Lack of immunosuppressive therapy impact assessment on determined subpopulations is one of the limitations of the study. Such analysis was not possible due to small size of the study group. It seems, however, that the possible impact of immunosuppressive therapy on the results was at least partially eliminated by comparing groups of different disease activity proven by well known indicator. This research was not designed as prospective cohort study. Its aim was to evaluate number of CD3^+^CD8^+^CD28^−^ and CD3^+^CD8^+^CD28^−^Foxp3^+^ subpopulations in one point in groups of different disease activity, and thus lack of follow-up can be considered a limitation of this study.

## 7. Conclusions

In conclusion, our analysis does not confirm expression of Foxp3 molecule in CD3^+^CD8^+^CD28^−^ cells which suggests nonsuppressive and nonregulative properties of the CD3^+^CD8^+^CD28^−^ subpopulation. Our data is the first study to indicate increase in percentage and absolute count of CD3^+^CD8^+^CD28^−^ lymphocytes along with an increase in disease activity. That indicates importance of CD3^+^CD8^+^CD28^−^ lymphocytes in the inflammatory process and suggests that the extension of the CD3^+^CD8^+^CD28^−^ subpopulation is associated with the exacerbation of the disease. Highlighting aspects of the immune imbalances and autoimmunity, the results of present study are a part of a discussion on the significance of CD3^+^CD8^+^CD28^−^ cells in the pathogenesis of SLE.

## Figures and Tables

**Table 1:** Characteristics of the study group in terms of age, gender, and disease activity measured by SLEDAI scale.

	SLEDAI	
SLEDAI score	≤5	>5
Group size	15	39
Mean SLEDAI	3.2	13
Median SLEDAI	4	12
Min–Max SLEDAI	0–5	6–28
Mean age	32.7 ± 9.1	37.9 ± 14.9
Median age	32	33
Sex	♀: 13 (86.7%) ♂: 2 (13.3%)	♀: 39 (100%) ♂: 0 (0%)

## Abbreviations

AAV: ANCA-associated vasculitis
ANCA: Antineutrophil cytoplasmic antibody
APC: Antigen presenting cell
CD: Cluster of differentiation
CTL: Cytotoxic T lymphocyte
FBS: Fetal bovine serum
Foxp3: Transcription factor forkhead box P3
HIV: Human immunodeficiency virus
HSP 27: Heat shock protein 27
LN: Lupus nephritis
MHC: Major histocompatibility complex
pAPCs: Professional antigen presenting cells
PBS: Phosphate buffered saline
rSLEDAI: Renal Systemic Lupus Erythematosus Disease Activity Index
SLE: Systemic lupus erythematosus
SLEDAI: Systemic Lupus Erythematosus Disease Activity Index
SSc: Systemic sclerosis
TCR: T-cell receptor.
